# Complement receptor 3-dependent engagement by *Candida glabrata* β-glucan modulates dendritic cells to induce regulatory T-cell expansion

**DOI:** 10.1098/rsob.230315

**Published:** 2024-05-29

**Authors:** Areerat Kunanopparat, Truc Thi Huong Dinh, Pranpariya Ponpakdee, Panuwat Padungros, Warerat Kaewduangduen, Kasirapat Ariya-anandech, Phawida Tummamunkong, Amanee Samaeng, Pannagorn Sae-ear, Asada Leelahavanichkul, Nattiya Hirankarn, Patcharee Ritprajak

**Affiliations:** ^1^ Department of Microbiology, Faculty of Dentistry, Center of Excellence in Integrative Immuno-Microbial Biochemistry and Bioresponsive Nanomaterials, Chulalongkorn University, Bangkok, Thailand; ^2^ Center of Excellence in Immunology and Immune-Mediated Diseases, Faculty of Medicine, Chulalongkorn University, Bangkok, Thailand; ^3^ Medical Microbiology Interdisciplinary Program, Graduate School, Chulalongkorn University, Bangkok, Thailand; ^4^ Department of Pathophysiology and Immunology, Faculty of Medicine, Can Tho University of Medicine and Pharmacy, Vietnam; ^5^ Department of Chemistry, Faculty of Science, Green Chemistry for Fine Chemical Production and Environmental Remediation Research Unit, Chulalongkorn University, Bangkok, Thailand; ^6^ Faculty of Dentistry, Oral Biology Research Center, Chulalongkorn University, Bangkok, Thailand; ^7^ Department of Microbiology, Faculty of Medicine, Center of Excellence in Translational Research in Inflammation and Immunology (CETRII), Chulalongkorn University, Bangkok, Thailand; ^8^ Department of Microbiology, Faculty of Dentistry, Chulalongkorn University, Bangkok, Thailand

**Keywords:** *Candida glabrata*, immune modulation, dendritic cell, regulatory T-cell, complement receptor 3

## Abstract

*Candida glabrata* is an important pathogen causing invasive infection associated with a high mortality rate. One mechanism that causes the failure of *Candida* eradication is an increase in regulatory T cells (Treg), which play a major role in immune suppression and promoting *Candida* pathogenicity. To date, how *C. glabrata* induces a Treg response remains unclear. Dendritic cells (DCs) recognition of fungi provides the fundamental signal determining the fate of the T-cell response. This study investigated the interplay between *C. glabrata* and DCs and its effect on Treg induction. We found that *C. glabrata* β-glucan was a major component that interacted with DCs and consequently mediated the Treg response. Blocking the binding of *C. glabrata* β-glucan to dectin-1 and complement receptor 3 (CR3) showed that CR3 activation in DCs was crucial for the induction of Treg. Furthermore, a ligand–receptor binding assay showed the preferential binding of *C. glabrata* β-glucan to CR3. Our data suggest that *C. glabrata* β-glucan potentially mediates the Treg response, probably through CR3-dependent activation in DCs. This study contributes new insights into immune modulation by *C. glabrata* that may lead to a better design of novel immunotherapeutic strategies for invasive *C. glabrata* infection.

## 1. Introduction

An opportunistic yeast, *Candida glabrata*, is the second most frequent cause of invasive candidiasis, which is often nosocomial [1,2]. The rapid progression of invasive candidiasis caused by *C. glabrata* is associated with substantial morbidity and mortality, perhaps owing to its inherent low susceptibility to anti-fungal medications, azoles, polyenes and echinocandins [2,3]. Although *C. glabrata* does not have the morphological flexibility that is a crucial virulence determinant of *C. albicans*, *C. glabrata* is a successful human pathogen because of its immune evasion ability [4,5].

A mechanism causing the failure of host protective immunity and an increase in *Candida* pathogenicity in systemic candidiasis is an increased response of regulatory T cells (Treg) [6]. Treg are a reciprocal T-cell subset of the antifungal T helper 17 (Th17) [7]. β-glucan is the most abundant polysaccharide of the inner skeleton of the *Candida* cell wall, and it plays a pivotal role in the induction of the host defence against *Candida* infection [8]. Recognition of *Candida* β-glucan by dectin-1 is crucial for the induction of antifungal Th17 immunity [9,10]. Treg expansion is mediated by the recognition of *C. albicans* via Toll-like receptor (TLR)-2-Toll/IL-1R domain-containing adaptor-inducing IFN (TRIF) signalling in antigen-presenting cells [11,12]. Dectin-1 is also important for the eradication of systemic *C. glabrata* infection [13,14]; however, the induction of Treg by *C. glabrata* remains to be determined.

Complement receptor 3 (CR3 or Mac-1) is composed of the heterodimer of α_M_ (CD11b) and β_2_ (CD18) integrin [15]. CR3 primarily recognizes *Candida* β-glucans via its lectin domain [15,16], which leads to the induction of an immune response to *Candida* infection [16–19]. However, the function of CR3 varies, probably depending on its binding sites and the characteristics of its ligands [20]. Recent evidence has shown a role of CR3 in immune suppression mediated by β-glucan derived from *C. albicans* [19]. Additionally, CR3 is involved in the induction of immune tolerance [20], and CR3 activation in dendritic cells (DCs) suppresses their immunostimulatory function [21].

DCs are the most potent antigen-presenting cells that are central to immune activation and immune tolerance [22]. DCs abundantly express pattern-recognition receptors (PRRs), such as C-type lectin receptors and Toll-like receptors, which play pivotal roles in sensing invading fungi. The interaction between PRRs and fungal-derived pathogen-associated molecular patterns (PAMPs) enables the functional versatility of DCs, which orchestrates T-cell fate and function [23]. The induction of Treg also requires the signal transduction carried by DCs [24]. At present, little is known regarding the interplay between *C. glabrata* and DCs. Therefore, this study aimed to investigate the role of *C. glabrata* β-glucan in the immune modulation of DCs via its specific receptors, dectin-1 and CR3. We also examined the effect of *C. glabrata* β-glucan-DC interactions on T-cell responses. A better understanding of the host immune response to *C. glabrata* might help develop new or improved approaches to control and treat invasive fungal infection.

## 2. Results

### 2.1. High-dose *C. glabrata* infection mediates regulatory T-cell expansion and high interleukin-10 production

**Figure 1.**
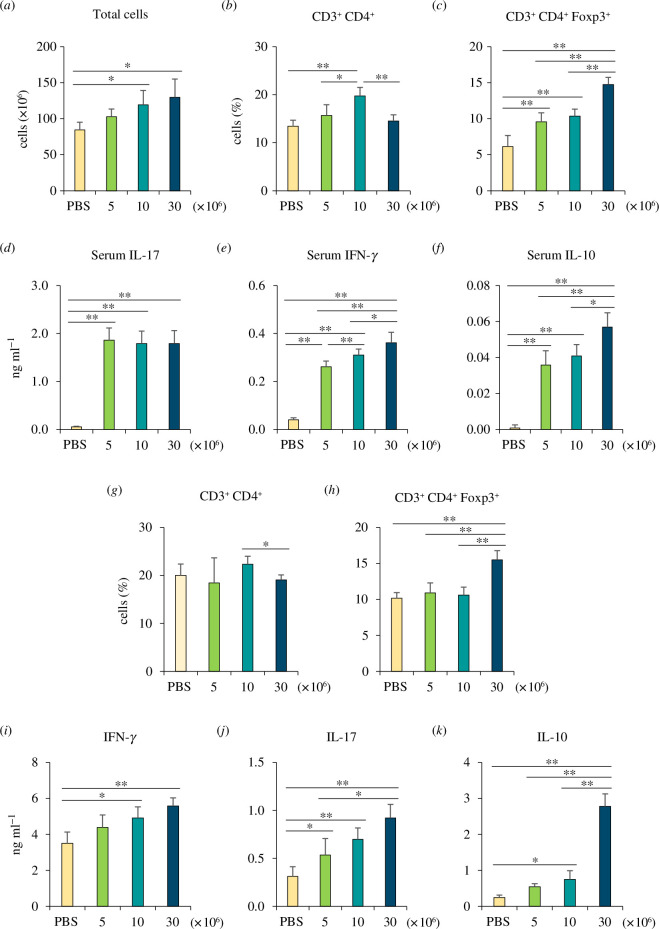
Systemic *C. glabrata* infection and *ex vivo* restimulation. Mice were systemically infected with 5 × 10^6^, 10 × 10^6^ and 30 × 10^6^ of *C. glabrata* yeast cells. Serum and spleens were collected on day 7 post-infection. In fresh splenocytes, (*a*) the total number of splenocytes was counted, and (*b*) CD3^+^CD4^+^ and (*c*) CD4^+^FoxP3^+^ T cells were assessed by flow cytometry. (*d*–*f*) Serum cytokines were quantified. Splenocytes were re-stimulated *ex vivo* with immobilized anti-CD3 for 48 h, and (*g*) CD3^+^CD4^+^ and (*h*) CD4^+^FoxP3^+^ T cells were assessed by flow cytometry. (*i*,*j*) Cytokines from the supernatant of anti-CD3-stimulated splenocytes (*n* = 6). *p < 0.05, **p < 0.01.

To examine the alteration in immune responses to systemic *C. glabrata* infection, mice were intravenously inoculated with various doses of *C. glabrata* and a splenic T-cell population, and serum cytokines were determined at day 7 post-infection (figure 1 and electronic supplementary material, figure S1). Total splenic cell numbers were increased in accordance with the increased doses of *C. glabrata,* while the proportion of splenic CD3^+^CD4^+^ T cells was notably increased in medium-dose *C. glabrata*-infected mice (10 × 10^6^ cells) (figure 1*a,b*). However, the proportion of CD3^+^CD4^+^ T cells was diminished in high-dose *C. glabrata*-infected mice (30 × 10^6^ cells) (figure 1*b*). Interestingly, the splenic Foxp3^+^ Treg population was significantly increased in a dose-dependent fashion. This population was the most abundant in high-dose *C. glabrata*-infected mice, which was consistent with the decreased CD3^+^CD4^+^ T cells in this high-dose infected group (figure 1*c* and electronic supplementary material, figure S1*a*).

Serum interleukin (IL)-17 concentrations were significantly increased upon *C. glabrata* infection, but IL-17 concentrations were not different among the different doses of *C. glabrata* (figure 1*d*). Elevated serum interferon (IFN)-γ and IL-10 concentrations were associated with the increased doses of *C. glabrata* (figure 1*e,f*). Notably, serum IL-10 concentrations in high-dose *C. glabrata*-infected mice (figure 1*f*) were substantially elevated in tandem with the increase in Foxp3^+^ Treg (figure 1*c*).

Splenocytes from all groups were restimulated *ex vivo* with immobilized anti-CD3 to determine T-cell specific responses (figure 1*g,k* and electronic supplementary material, S1*b*). The proportion of restimulated CD3^+^CD4^+^ T cells was not changed in the low-dose and medium-dose groups, but that of CD3^+^CD4^+^ T cells was reduced in splenocytes from high-dose *C. glabrata*-infected mice (figure 1*g*). Foxp3^+^ Treg were apparently increased in the restimulated splenocytes from high-dose *C. glabrata* infection (figure 1*h* and electronic supplementary material, figure S1*b*). Additionally, IFN-γ, IL-17 and IL-10 concentrations were detected in the culture supernatant of the restimulated splenocytes. All cytokines were increased in a dose-dependent fashion (figure 1*i*–*k*). However, IL-10 concentrations were markedly augmented in splenocytes from high-dose *C. glabrata*-infected mice (figure 1*k*). Taken together, these results suggest that systemic *C. glabrata* infection induces the immunosuppressive response by promoting Treg expansion and IL-10 production.

### 2.2. *Candida glabrata* β-glucan induces antigen-specific Treg expansion and augmented IL-10 production

**Figure 2.**
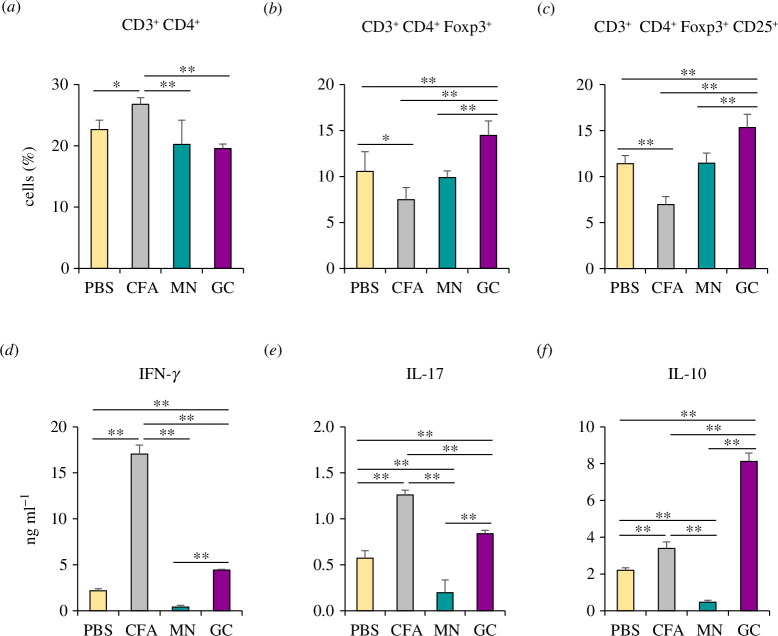
*In vivo* immunization of *C. glabrata* mannan and β-glucan. Mice were subcutaneously immunized with phosphate-buffered saline, CFA/IFA, *C. glabrata* mannan (50 µg per 1 g of body weight), or *C. glabrata* β-glucan (50 µg per 1 g of body weight) along with 30 µg OVA on days 0 and 7. On day 14, LN cells were restimulated *ex vivo* with 250 µg ml^−1^ of OVA for 72 h. (*a*) CD3^+^CD4^+^, (*b*) CD3^+^CD4^+^FoxP3^+^ and (*c*) CD3^+^CD4^+^FoxP3^+^CD25^+^ T cells were assessed by flow cytometry. (*d*–*f*) Cytokines from supernatant of restimulated LN cells (*n* = 5). *p < 0.05, **p < 0.01. CFA, CFA/IFA; MN, mannan; GC, β-glucan.

Mannans and β-glucans are major carbohydrate constituents in the *Candida* cell wall and they play critical roles in immune modulation [8,25]. Therefore, we examined whether these components from *C. glabrata* are involved in eliciting the Treg response. *C. glabrata* mannan or β-glucan was mixed with an immunogenic antigen, oval chicken albumin (OVA), and was subcutaneously administered to mice on days 0 and 7. In this experiment, complete Freund’s adjuvant (CFA) was used as the positive control because this adjuvant potentially activates the immune response. On day 14, lymph node (LN) cells from immunized mice were restimulated *ex vivo* with OVA and a T-cell population, and the cytokine profile was analysed (figure 2). CFA effectively enhanced antigen-specific CD4 T-cell expansion, while *C. glabrata* mannan and β-glucan did not (figure 2*a*). In agreement with the increased number of CD3^+^CD4^+^ T cells, the number of antigen-specific Foxp3^+^ and Foxp3^+^CD25^+^ Treg was diminished in the CFA immunized group (figure 2*b,c*, electronic supplementary material, figure S2). In contrast, *C. glabrata* β-glucan, but not mannan, potentially induced Foxp3^+^ and Foxp3^+^CD25^+^ Treg expansion (figure 2*b,c*).

IFN-γ, IL-17 and IL-10 production was also detected in the supernatant of the antigen-restimulated LN cells (figure 2*d*–*f*). In accordance with T-cell responses (figure 2*a,b*), LN cells from *C. glabrata* mannan-immunized mice did not produce any cytokines, while LN cells from *C. glabrata* β-glucan-immunized mice markedly produced IL-10, but only slightly produced IFN-γ and IL-17. These data suggest that *C. glabrata* β-glucan plays a crucial role in Treg induction and IL-10 production in systemic *C. glabrata* infection.

### 2.3. DC responses are mainly modulated by *C. glabrata* β-glucan

**Figure 3.**
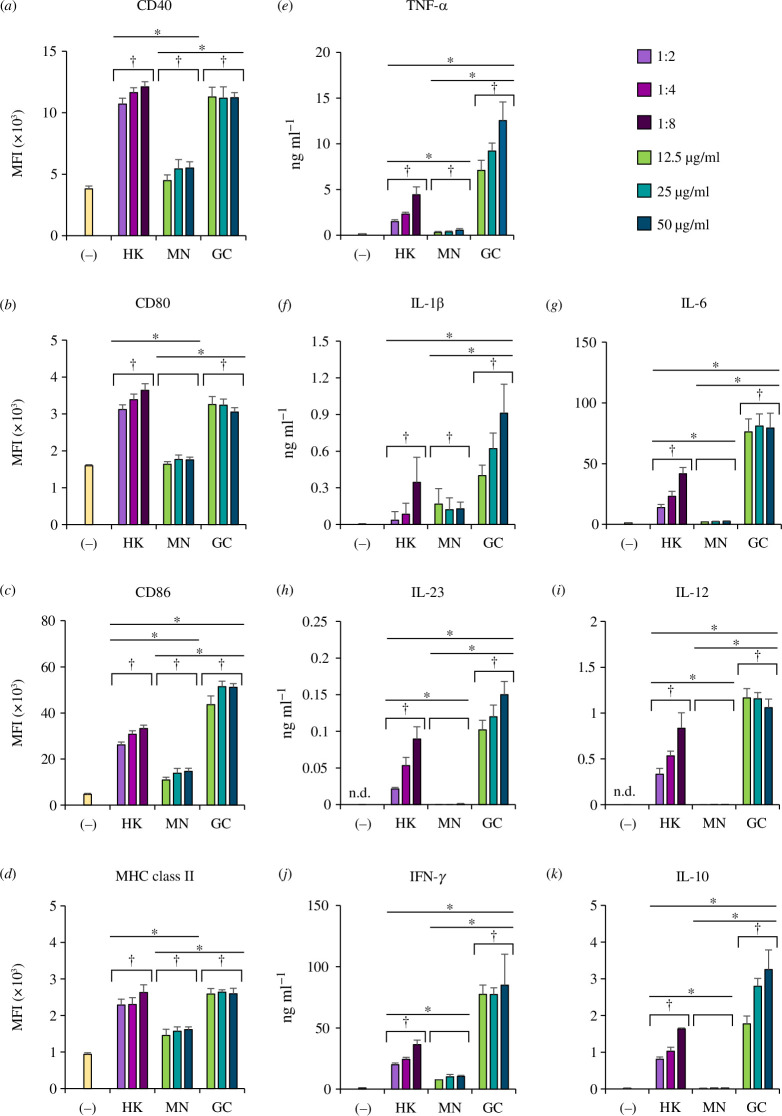
Stimulation of DCs with heat-killed yeast, mannan and β-glucan of *C. glabrata.* BMDCs were stimulated with heat-killed *C. glabrata* (at DC  /yeast ratios of 1 : 2, 1 : 4 and 1 : 8), *C. glabrata* mannan (12.5, 25 and 50 µg ml^−1^), and *C. glabrata* β-glucan (12.5, 25 and 50 µg ml^−1^) for 24 h. (*a*–*d*) DC maturation markers were assessed by flow cytometry. MFI was determined by a histogram analysis. (*e*–*k*) Cytokine production was quantified by ELISA. The data were analysed using two-way analysis of variance (*n* = 5). ^†^ *p* < 0.05 compared with unstimulated BMDCs, *p < 0.05, **p < 0.01. MFI, mean fluorescence intensity; (−), unstimulated BMDCs; HK, heat-killed yeast; MN, mannan; GC, β-glucan.

DCs play a central role in T-cell differentiation, and the interaction between DCs and mannans and β-glucans in the C*andida* cell wall is important to modulate the host immune responses [26]. Therefore, we compared the responses of bone marrow-derived DCs (BMDCs) to β-glucan, mannan and heat-killed *C. glabrata* yeasts that could not produce all secretory molecules, and allowed the cell wall components (mainly composed of mannan and β-glucan) to interact with DCs. DC maturation and cytokine production were determined at 24 h post-stimulation by flow cytometry and an enzyme-linked immunosorbent assay (ELISA), respectively (figure 3 and electronic supplementary material, figure S2). Heat-killed yeasts and β-glucan strongly induced the expression of DC maturation markers (CD40, CD80, CD86 and MHC class II) to similar levels, while mannan slightly activated DCs (figure 3*a*–*d* and electronic supplementary material, figure S2*a*,*b*). β-Glucan and, to a lesser extent, heat-killed yeasts potentially activated BMDCs to produce all cytokines, including IL-10, while mannan failed to stimulate cytokine production in BMDCs (figure 3*e*–*k*). Additionally, these different DC responses induced by heat-killed yeasts, mannan and β-glucan were not associated with DC viability because a cell viability test showed no difference among all stimulations (electronic supplementary material, figure S2*c*). Therefore, *C. glabrata* β-glucan, but not mannan, may be the main component that modulates DC responses.

### 2.4. DCs differentially respond to *C. glabrata and C. albicans* β-glucan

**Figure 4.**
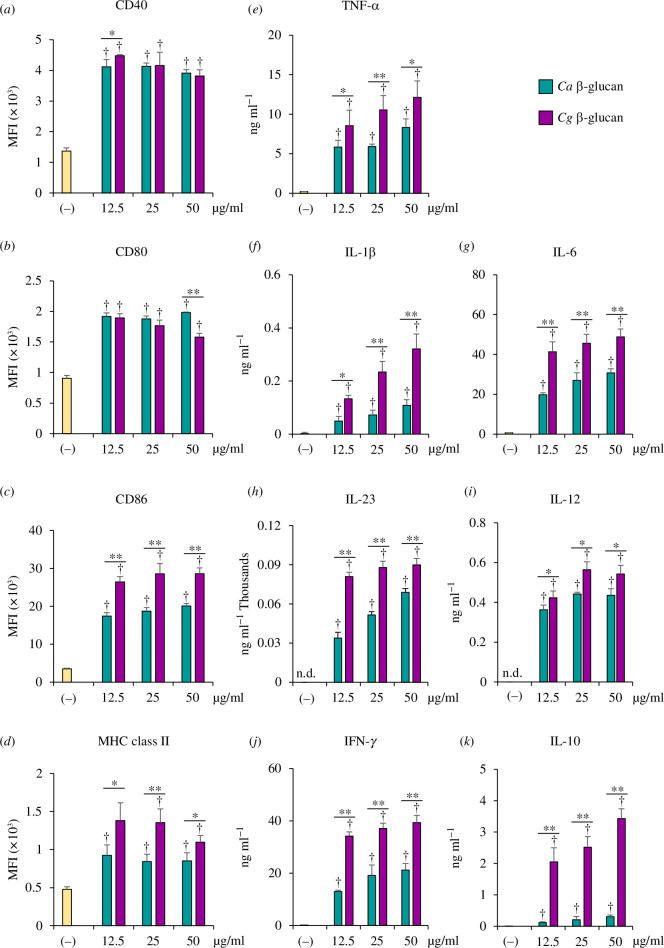
Stimulation of DCs with *C. albicans* and *C. glabrata* β-glucan. BMDCs were stimulated with 12.5, 25 and 50 µg ml^−1^ of β-glucan isolated from *C. albicans* and *C. glabrata* for 24 h. (*a*–*d*) DC maturation markers were assessed by flow cytometry. MFI was determined by a histogram analysis. (*e*–k) Cytokine production in the culture supernatant was quantified by ELISA (*n* = 5). ^†^ *p*<0.05 compared with unstimulated BMDCs, *p < 0.05, **p < 0.01. MFI, mean fluorescence intensity; (−), unstimulated BMDCs; *Ca*, *C. albicans*; *Cg*, *C. glabrata*.

*C. albicans* β-glucan enhances DC immunogenicity and consequently mediates adaptive immunity that eradicates *C. albicans* infection [27]. However, little is known about the DC response to *C. glabrata* β-glucan. BMDCs were stimulated with various doses of *C. glabrata* β-glucan and compared with *C. albicans* β-glucan, and BMDC maturation and cytokine production were determined (figure 4 and electronic supplementary material, figure S3). The expression levels of CD40 and CD80 in BMDCs stimulated with *C. glabrata* β-glucan and *C. albicans* β-glucan were increased to the same extent (figure 4*a*, *b*). However, *C. glabrata* β-glucan induced higher CD86 and MHC class II expression in BMDCs (figure 4*c,d*). Overall, BMDCs stimulated with *C. glabrata* β-glucan showed higher cytokine production than BMDCs stimulated with *C. albicans* β-glucan (figure 4*e*–*k*). *C. albicans* β-glucan induced a low amount of IL-10 production in BMDCs, but *C. glabrata* β-glucan induced substantial IL-10 production (approximately 10-fold difference) (figure 4*k*). Furthermore, all doses of both *Candida* β-glucans did not affect DC viability (electronic supplementary material, figure S3*c*). These data suggest that DCs respond differently to β-glucan from two different *Candida* species, and *C. glabrata* β-glucan has a greater ability to promote IL-10 production in DCs.

### 2.5. *Candida glabrata* β-glucan interacts with dectin-1 and CR3 in DCs in a different manner

**Figure 5.**
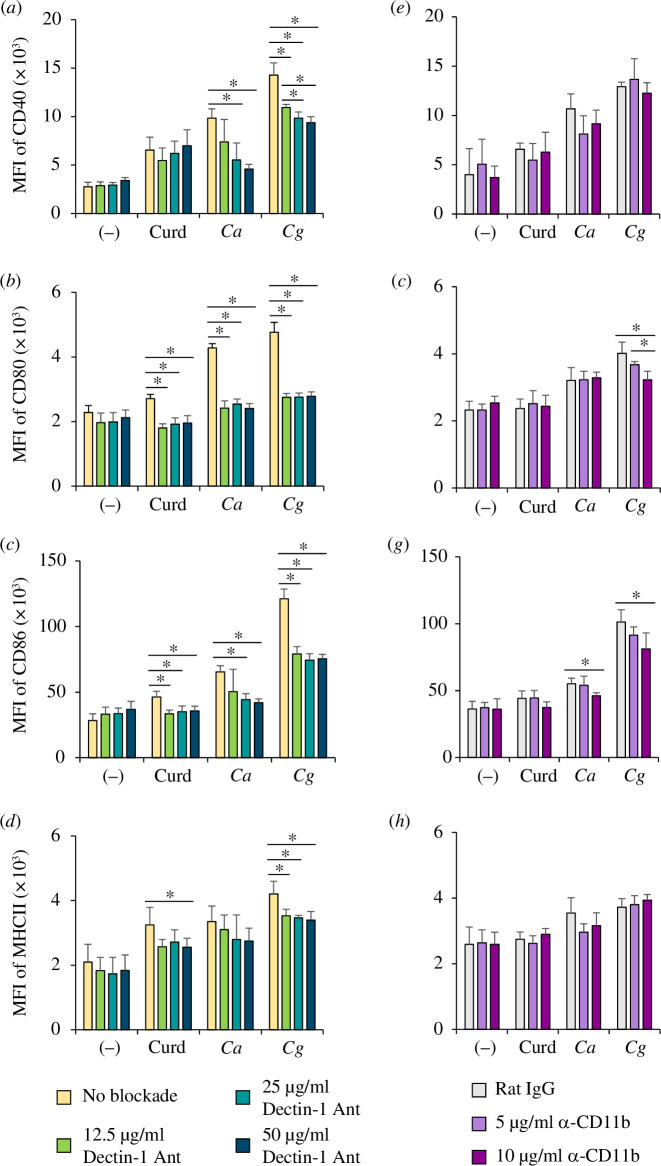
Dectin-1-dependent and CR3-dependent DC maturation. BMDCs were pre-treated with a dectin-1 antagonist (12.5, 25 and 50 µg ml^−1^) or anti-CD11b (5 and 10 µg ml^−1^), and then the cells were stimulated with 25 µg/ml of curdlan and β-glucan isolated from *C. albicans* and *C. glabrata* for 24 h. In the negative control, BMDCs were unblocked or pre-treated with rat IgG (10 µg ml^−1^). Analysis of (*a*–*d*) dectin-1 blockade and (*e*, *f*) CR3 blockade. DC maturation markers were assessed by flow cytometry. MFI was determined by a histogram analysis (*n* = 5). *p < 0.05, **p < 0.01. MFI, mean fluorescence intensity; (−), unstimulated BMDCs; Curd, curdlan; *Ca*, *C. albicans; Cg*, *C. glabrata*.

Dectin-1 and CR3 (CD11b/CD18) play a pivotal role in the host defence mechanism through the recognition of *Candida* β-glucans [9,10,15,16]. To investigate how DCs respond to *C. glabrata* β-glucan via dectin-1 and CR3, BMDCs were pre-incubated with a dectin-1 antagonist and anti-CD11b mAb before *Candida* β-glucan stimulation. Anti-CD11b was used to block the binding of β-glucans to CR3 because CD11b contains a lectin domain that ligates to β-glucans [28]. Curdlan was used as a positive control because it is a well-known ligand that can bind to dectin-1 and CR3 [29,30]. The blockade of dectin-1 in BMDCs stimulated with curdlan, *C. albicans* β-glucan and *C. glabrata* β-glucan significantly inhibited the expression of the DC maturation markers CD40, CD80, CD86 and MHC class II at early stage (24 h) and late stage (48 h) of DC maturation in response to β-glucan stimulation (ffigure 5*a*–*d*, and electronic supplementary material, figures S4 and S6*a*–*d*). Notably, the blockade of CR3-inhibited BMDC maturation in response to curdlan and *C. glabrata* β-glucan at 24 and 48 h after β-glucan stimulation, while this blockade barely inhibited the maturation of BMDCs stimulated with *C. albicans* β-glucan (figure 5*e*–*h* , and electronic supplementary material, figure S6*e*–*h*). Furthermore, dectin-1 blockade inhibited DC maturation upon *C. glabrata* β-glucan and *C. albicans* β-glucan stimulation more than CR3 blockade (figure 5 and electronic supplementary material, figure S6). Additionally, the inhibition of dectin-1 impacted the early stage of DC maturation, whereas the inhibition of CR3 affected the late stage of DC maturation.

**Figure 6.**
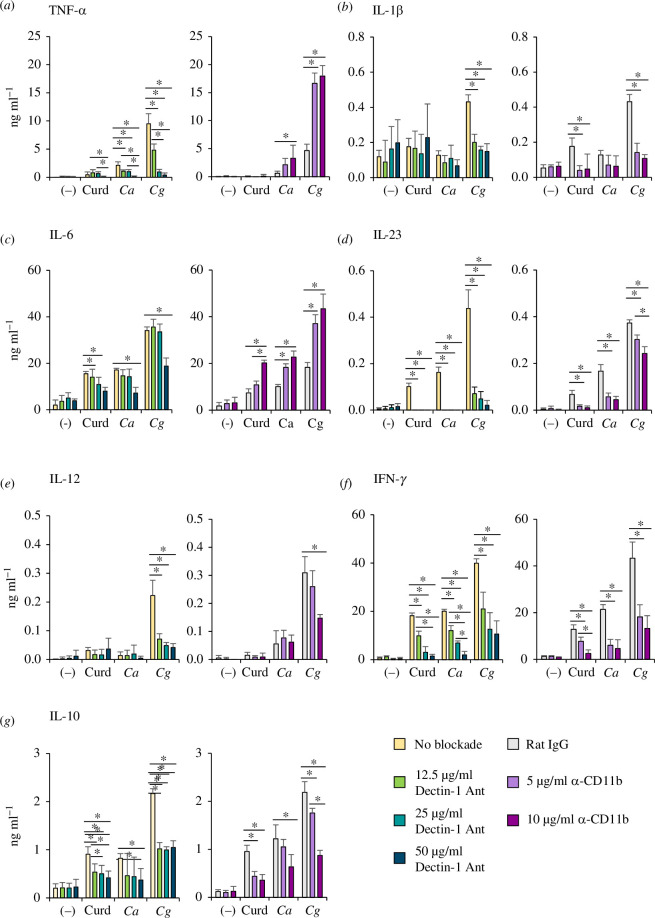
Dectin-1-dependent and CR3-dependent cytokine production in DCs. BMDCs were pre-treated with a dectin-1 antagonist (12.5, 25 and 50 µg ml^−1^) or anti-CD11b (5 and 10 µg ml^−1^), and then the cells were stimulated with 25 µg ml of curdlan and β-glucan isolated from *C. albicans* and *C. glabrata* for 24 h. In the negative control, BMDCs were unblocked or pre-treated with rat IgG (10 µg ml^−1^). (*a*–*f*) Cytokine production in the culture supernatant was quantified by ELISA (*n* = 5). †*p* < 0.05 compared with unstimulated BMDCs, *p < 0.05, **p < 0.01. (–), unstimulated BMDCs; *Ca*, *C. albicans*; *Cg*, *C. glabrata*.

The cytokine profile in the culture supernatant of BMDCs was also determined. Blockade of dectin-1 and CR3 in curdlan-, *C. albicans* β-glucan- and *C. glabrata* β-glucan-stimulated BMDCs showed different inhibition levels of cytokine production (figure 6 and electronic supplementary material, figure S7). CR3 blockade significantly enhanced TNF-α and IL-6 production, while dectin-1 blockade diminished the production of these cytokines in all stimulation conditions (figure 6*a*,*c* figure 6*a,c* and electronic supplementary material, figure S7*a*,*c*). To ensure that the dectin-1 antagonist and anti-CD11b treatments did not have any toxicity on the BMDCs, which could potentially affect their responses, we conducted a cell viability test. Our results indicated that both treatments had no significant impact on the viability of the DCs (electronic supplementary material, figure S9). These data suggest variation in the interaction between the distinct *Candida* β-glucans and their receptors, dectin-1 and CR3. Additionally, *C. glabrata* β-glucan interacts with dectin-1 and CR3 in a different manner.

### 2.6. *Candida glabrata* β-glucan mediates Treg expansion via CR3 on DCs

**Figure 7.**
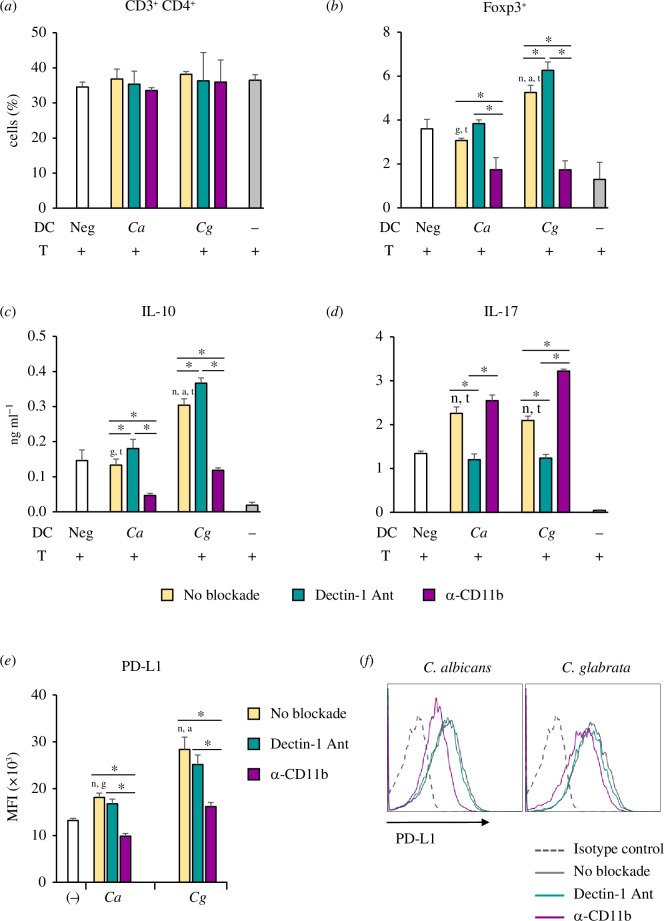
Contribution of dectin-1 versus CR3 blockade to the DC-mediated Treg response. BMDCs were pre-treated with a dectin-1 antagonist (25 µg ml^−1^) or anti-CD11b (10 µg ml^−1^), and then the cells were stimulated with 25 µg ml^−1^ of β-glucan isolated from *C. albicans* and *C. glabrata* for 24 h. The stimulated BMDCs were co-cultured with murine splenic T cells at a DC:T-cell ratio of 1:10 in the presence of soluble anti-mouse CD3 monoclonal antibody (30 ng ml^−1^) for 48 h. (*a*) CD3^+^CD4^+^ and (*b*) CD4^+^FoxP3^+^ T cells were assessed by flow cytometry. The concentrations of (*c*) IL-10 and (*d*) IL-17 in the supernatant of DC–T-cell co-culture were determined by ELISA. (*e*) BMDCs were pre-treated with a dectin-1 antagonist (25 µg ml^−1^) or anti-CD11b (10 µg ml^−1^), and then the cells were stimulated with 25 µg ml^−1^ of β-glucans isolated from *C. albicans* and *C. glabrata* for 24 h. PD-L1 expression in BMDCs was investigated by flow cytometry. MFI was determined by a histogram analysis (*n* = 3). (*f*) Histogram analysis of PD-L1 expression. ^n^ *p* < 0.05 compared with unstimulated BMDCs, ^t^ *p* < 0.05 compared with T cells alone, ^a^ *p* < 0.05 compared with *C. albicans* β-glucan-stimulated BMDCs, ^g^ *p* < 0.05 compared with *C. glabrata* β-glucan-stimulated BMDCs, *p < 0.05. MFI, mean fluorescence intensity; Neg, unstimulated BMDCs; Ca, *C. albicans* β-glucan-stimulated BMDCs; Cg, *C. glabrata* β-glucan-stimulated BMDCs; +, presence; –, absence.

After demonstrating the *in vivo* effects of *C. glabrata* β-glucan on Treg expansion (figure 2), we next examined whether the engagement of *C. glabrata* β-glucan to its receptors, dectin-1 and CR3, in DCs affects Treg differentiation. *In vitro* DC–T-cell co-cultures were performed in the presence of a soluble anti-CD3 mAb. In this experimental setting, the Fc portion of anti-CD3 mAb binds to the Fc receptor on DCs, while the Fab portion binds to CD3 on T cells. With this interaction, T-cell receptor (TCR) signalling is activated [31,32]. In parallel, the co-signals and cytokines were derived from BMDCs that were stimulated with *C. albicans* and *C. glabrata* β-glucan in the presence or absence of a dectin-1 antagonist or anti-CD11b mAb (figure 7). Forty-eight hours after the co-cultures, CD3^+^CD4^+^ and CD3^+^CD4^+^Foxp3^+^ T cells were identified using flow cytometry (figure 7*a* ). Although the proportion of CD3^+^CD4^+^ T cells was not different in all conditions (figure 7*a*), the Foxp3^+^ Treg population was notably increased when BMDCs were stimulated with *C. glabrata* β-glucan (figure 7*b*). Intriguingly, the Foxp3^+^ Treg population was increased when dectin-1 on BMDCs was blocked, but this population was markedly decreased upon CR3 blockade (figure 7*b*).

An immunosuppressive cytokine, IL-10 and an anti-fungal cytokine, IL-17, in the supernatant of the DC–T-cell co-cultures were also determined. Consistent with the Treg responses (figure 7*b*), IL-10 concentrations were increased upon dectin-1 blockade and diminished when CR3 on BMDCs was blocked (figure 7*c*). In contrast, concentrations of IL-17, representing Th17, which is a reciprocal development pathway of Treg [33], were decreased when dectin-1 on BMDCs was blocked and it was increased upon CR3 blockade (figure 7*d*). Blockades of both receptors in BMDCs stimulated with *C. albicans* and *C. glabrata* β-glucan produced similar Foxp3^+^ Treg and cytokine responses (figure 7*a*–*d*).

To observe the impact of dectin-1 and CR3 ligation in DCs on activated T cells along with Treg, splenic T cells were isolated and co-cultured with BMDCs in the presence of soluble anti-CD3 as described above. At 48 h after the co-culture, T-cell proliferation was determined by flow cytometric analysis (electronic supplementary material, figure S10). CD3^+^CD4^+^CD25^+^Foxp3^−^ activated T cells and CD3^+^CD4^+^ Foxp3^+^ Treg were gated, and then CFSE^low^ cells (proliferated cells) were analysed (electronic supplementary material, figure S10*a*). Consistently, T cells co-cultured with *C. glabrata* β-glucan-stimulated DCs showed the lower number of CD3^+^CD4^+^CD25^+^Foxp3^−^ activated T cells but had the increased number of CD3^+^CD4^+^Foxp3^+^ Treg. Blockade of CR3, but not dectin-1, in *C. glabrata* β-glucan-stimulated DCs increased the number of CD3^+^CD4^+^CD25^+^Foxp3^−^ activated T cells and decreased the number of CD3^+^CD4^+^Foxp3^+^ Treg (electronic supplementary material, figure S10*b,c*). These results suggested that the alteration in CD3^+^CD4^+^CD25^+^Foxp3^−^ activated T cells may be resulted from the suppressive activity of the Treg mediated by *C. glabrata* β-glucan-stimulated DCs in a CR3-dependent manner.

To evaluate the effect of dectin-1 and CR3 ligation in DCs on activated T cells and Treg, splenic T cells were isolated and co-cultured with BMDCs in the presence of soluble anti-CD3. T-cell proliferation was assessed after 48 h of co-culture by flow cytometric analysis. Activated T cells (CD3^+^CD4^+^CD25^+^Foxp3^−^) and Treg (CD3^+^CD4^+^Foxp3^+^) were identified and analysed for CFSE^low^ cells (proliferated cells) (electronic supplementary material, figure S10*a*). T cells co-cultured with *C. glabrata* β-glucan-stimulated DCs displayed a lower number of activated T cells but an increased number of Treg. Blockade of CR3, but not dectin-1, in *C. glabrata* β-glucan-stimulated DCs augmented the number of activated T cells and reduced the number of Treg (electronic supplementary material, figure S10*b,c*). These findings suggest that the decrease in activated T cells may be owing to the suppressive activity of Treg mediated by *C. glabrata* β-glucan-stimulated DCs in a CR3-dependent manner.

Dectin-1 and CR3 were involved in IL-10 production (figure 6*g* and electronic supplementary material, figure S7G), but how CR3 activation is associated with Treg expansion is unclear. Program death ligand 1 (PD-L1) expression in DCs is essential for the development and suppressive function of Treg [34,35]. Therefore, we investigated whether PD-L1 expression in *Candida* β-glucan-stimulated BMDCs is associated with Treg expansion. Dectin-1 or CR3 on BMDCs was blocked before *Candida* β-glucan stimulation, and PD-L1 expression was evaluated. Augmented PD-L1 expression was observed in BMDCs stimulated with *C. glabrata* β-glucan (figure 7*e*), and blockade of CR3, but not dectin-1, markedly reduced PD-L1 expression in BMDCs (figure 7*e,f* ). Additionally, treatment with dectin-1 antagonist and anti-CD11b in the absence of β-glucan did not affect PD-L1 expression (electronic supplementary material, figure S11). These results indicated that alteration of Treg differentiation in response to *C. glabrata* β-glucan-stimulated BMDCs (figure 7*b*) may depend on a β-glucan-CR3 interaction.

### 2.7. Morphology and structural feature of *C. glabrata* β-glucans

**Figure 8.**
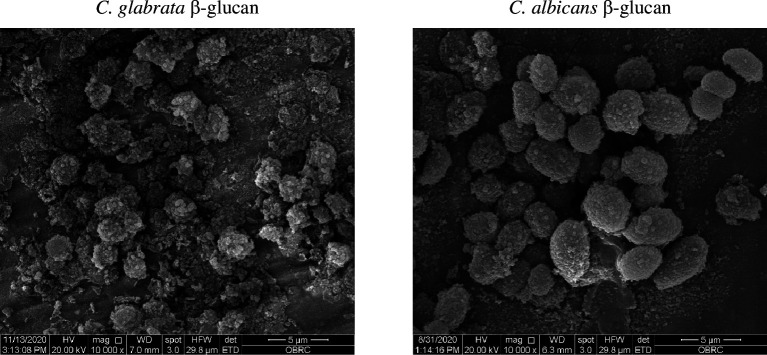
Morphological appearance of *C. albicans* and *C. glabrata* β-glucan. β-glucans of *C. albicans* and *C. glabrata* were observed by scanning electron microscopy with 10 000× magnification.

**Figure 9.**
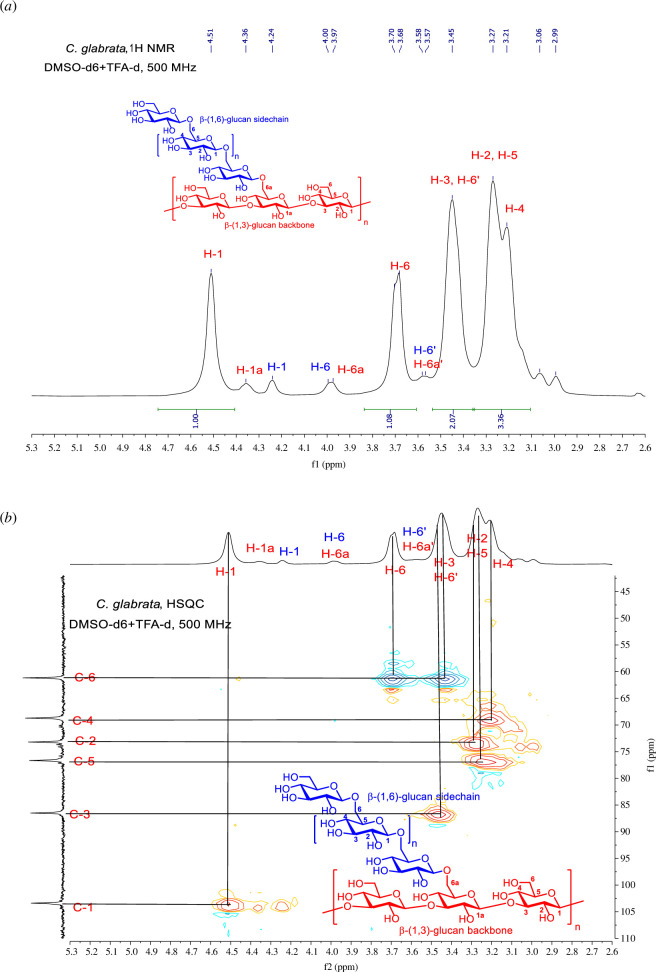
Structure analysis of *C. glabrata* β-glucan. (*a*)^1^H NMR spectrum of *C. glabrata* β-glucan (500 MHz, DMSO-d_6_+TFA-d_1_). (*b*) HSQC spectrum of *C. glabrata* β-glucan (500 MHz, DMSO-d_6_+TFA-d_1_). The spectrum indicates a highly homogeneous β-(1,3)-glucan with β-(1,6)-branching.

The morphological appearance and structure of *Candida* β-glucans are implicated in receptor binding activity [36–39]. Therefore, we observed the morphology and structure of *C. glabrata* β-glucan (figures 8 and 9, and electronic supplementary material, figure S8). The morphology of *C. glabrata* and *C. albicans* β-glucan was characterized by scanning electron microscopy. *C. glabrata* β-glucan formed a small spherical shape of approximately 2 µM in size, while *C. albicans* β-glucan formed a large oval shape of approximately 5 µM in size. Furthermore, *C. glabrata* β-glucan was a coarse and rough particle, but *C. albicans* β-glucan appeared as a dense fine particle (figure 8).

Characterization of the *C. glabrata* β-glucan structure was then carried out using nuclear magnetic resonance (NMR) spectroscopy (figure 9 and electronic supplementary material, figure S8). NMR characterization of carbohydrates [40–42], especially polysaccharides [39,43] can be challenging owing to various factors, such as poor solubility in deuterated solvents [39], the complexity of the carbohydrate structure [44–46], and signal overlap between protons of hydroxyl groups and the pyranose/furanose region [40,41]. Initially, *C. glabrata* β-glucan showed poor solubility in polar deuterated NMR solvents, such as dimethyl sulfoxide (DMSO)-d_6_, D_2_O and CD_3_OD. After several trials, adding deuterated trifluoroacetic acid (TFA-d_1_) to a solution of *C. glabrata* β-glucan in DMSO-d_6_ enhanced the solubility compared with using DMSO-d_6_ alone. Protonation and exchange of the deuterium ion from TFA-d_1_ with the hydroxyl proton additionally simplified the coupling signals between the hydroxyl and pyranose ring protons, thus, facilitating the interpretation of the ^1^H NMR spectra.

*Candida glabrata* β-glucan was comprehensively characterized previously by NMR analysis which showed that *C. glabrata* β-glucan consists of a β-(1,3)-glucan backbone and β-(1,6)-glucan sidechains [47]. In our study, satisfactory NMR spectra were obtained from *C. glabrata* β-glucan at room temperature. Two sets of ^1^H NMR signals were present. High- and low-intensity signals represented pyranose protons on the β-(1,3)-glucan backbone and β-(1,6)-glucan sidechain, respectively (figure 9*a*). In the β-(1,3)-glucan backbone (high intensity; red signals), H-1 was the most downfield signal and was found at 4.51 ppm, followed by H-6 at 3.68–3.70 ppm, H-3/H-6′ at 3.45 ppm, H-2/H-5 at 3.27 ppm and H-4 at 3.21 ppm. In the β-(1,6)-glucan sidechain (low intensity; blue signals), the anomeric proton H-1 appeared at 4.24 ppm, and H-6 and H-6′ appeared at 4.00 and 3.58 ppm, respectively. Additionally, ^1^H NMR signals of the branched glucose unit in the backbone were distinguished from the remainder of the glucose units (without branching) by the signals of H-1a at 4.36 ppm, and H-6a and H-6a′ at 3.97 and 3.57 ppm, respectively (low intensity; red signals).

^13^C NMR measurement of *C. glabrata* β-glucan also showed all six signals of the glucose units in the β-(1,3)-glucan backbone as follows: C-1 (β-anomeric carbon), C-3, C-5, C-2, C-4 and C-6 were at 103.4, 86.5, 76.7, 73.2, 68.7 and 61.2 ppm, respectively (electronic supplementary material, figure S8*a*). The correlated spectroscopy (COSY) homonuclear NMR experiment (^1^H-C-C-^1^H connectivity) (electronic supplementary material, figure S8*b*) and heteronuclear single quantum coherence (HSQC) NMR experiment (^1^H-^13^C connectivity) (figure 9*b*) also supported the proposed structure of *C. glabrata* β-glucan as a β-(1,3)-glucan backbone with a β-(1,6)-glucan sidechain.

### 2.8. *C. glabrata* β-glucan is bound to CR3 rather than *C. albicans* β-glucan

**Figure 10.**
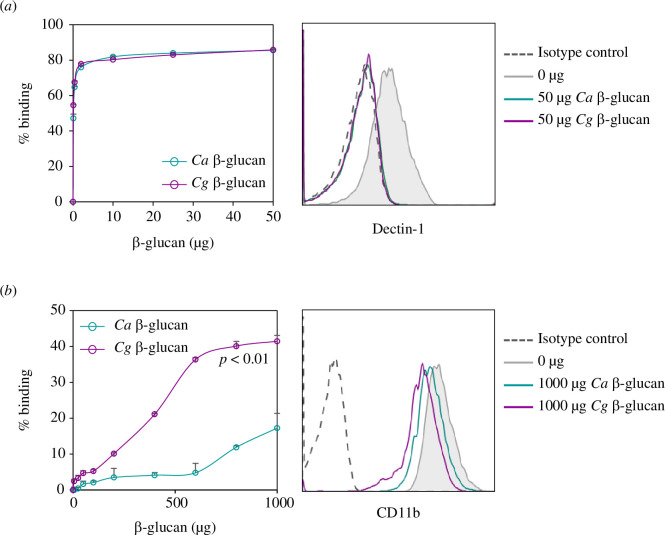
Binding of *C. albicans* and *C. glabrata* β-glucan to dectin-1 and CR3. RAW264.7 cells were incubated with various amounts of *C. albicans* or *C. glabrata* β-glucan for 20 min. After washing, the cells were stained with fluorescent-tagged anti-dectin-1 mAbs or fluorescent-tagged anti-CD11b mAbs. The expression of dectin-1 and CR3 was determined by flow cytometry (*n* = 3). The percentage binding was calculated as described in the Materials and methods section. (*a*) β-glucan and dectin-1 binding analysis and a flow cytometric histogram of dectin-1 expression. (*b*) β-glucan and CR3 binding analysis and a flow cytometric histogram of CR3 expression.

Because *C. glabrata* β-glucan showed a distinct morphology and structure, we next examined β-glucan-receptor binding properties by using a dectin-1- and CR3-expressing cell line, RAW264.7 macrophages (figure 10 and electronic supplementary material, figure S9). RAW264.7 cells were pre-incubated with various amounts of *C. albicans* or *C. glabrata* β-glucans, and then stained with fluorescent-tagged anti-dectin-1 and anti-CR3 monoclonal antibodies (mAbs). The results were determined by flow cytometric analysis (electronic supplementary material, figure S9). The binding of *C. glabrata* β-glucans to dectin-1 was similar to that of *C. albicans* β-glucan (figure 10*a*), while *C. glabrata* β-glucans bound to CR3 greater than *C. albicans* β-glucan (figure 10*b*).

## 3. Discussion

Studies have shown the crucial role of cell wall β-glucan of *C. albicans* in the induction of anti-fungal immunity [8,48]. In this study, we found that the cell wall β-glucans of *C. glabrata* had different effects on DC immunity and subsequent T-cell responses. The role of cell wall β-glucan in the yeast form of *Candida* was investigated because *C. glabrata* is a nondimorphic yeast. *Candida* cell walls are mainly constructed by an outer mannan layer and inner β-glucan skeleton [8]. *C. glabrata* mannan was found to induce DC and macrophage activation and cytokine production [49], but *C. glabrata* mannan in our study lacked the ability to induce immune responses (figures 2 and 3). These differences may have resulted from the inter-strain variations of the mannan structure [50]. However, β-glucan is exposed in the cell wall of the yeast form. Therefore, β-glucan in the inner layer can interact with the host PRRs [51]. Accordingly, *C. glabrata* heat-killed yeast and β-glucan showed similar DC responses (figure 3).

Dectin-1 is a receptor that recognizes *Candida* β-glucans and it has a major role in inducing anti-fungal immunity [10]. The genetic ablation of dection-1 in mice leads to an increased susceptibility to systemic infections caused by various *Candida* species [13,52]. Dectin-1-dependent responses to cell wall β-glucans differ among *Candida* species [13,39]. We found that the interaction between dectin-1 on BMDCs and the distinct *Candida* β-glucan led to a variation in DC responses (figures 5 and 6, electronic supplementary material, figures S6 and S7). CR3 also recognizes yeast-derived β-glucan and plays a pivotal role in the host defence mechanism [16,20]. In this study, we found that the ligation between β-glucans from distinct *Candida* species and CR3 also had different effects on immune responses (figures 5–7).

Although there is no evidence showing the binding characteristics between β-glucan and CR3, several lines of evidence suggest that the variation in β-glucan structures and contents is a main factor that affects receptor recognition and immune responses [13,36,53]. NMR analysis of other and our previous publications has shown that β-glucan of the *C. albicans* yeast form is composed of β-(1,3)-glucan with β-(1,6)-branching [37,39,54]. In this study, we found that β-glucan of *C. glabrata* yeast was also composed of β-(1,3)-glucan with β-(1,6)-branching, which is consistent with a previous report [47] (figure 9 and electronic supplementary material, figure S8). However, the composition of β-(1,3)-glucan and β-(1,6)-glucan of both *Candida* species was different. β-(1,3)-glucan of *C. glabrata* was homogeneous and uniform (figure 9 and electronic supplementary material, figure S8). Additionally, a high-field NMR analysis showed that *C. glabrata* β-glucan contained a larger amount of β-(1,6)-glucan than *C. albicans* [37,39,47,54]. Curdlan consists entirely of (1→3)-β-d-glucosidic linkages [55]. Blockade of dectin-1 and CR3 affected BMDC response to curdlan differently than Candida β-glucan. (electronic supplementary material, figures S7 and S8). The data have confirmed that β-glucan structures and contents are significant in the response of DC. The different particle sizes of β-glucans may be partly responsible for the variation in immune responses [38]. As shown by a morphological analysis, *C. albicans* and *C. glabrata* β-glucan differed in size and shape (figure 8).

The particulate or immobilized form of β-glucan is required for inducing signal transduction via dectin-1, while the soluble and particulate forms of fungal β-glucan can activate CR3 [18,19,56]. In this study, we used the particulate form of *Candida* β-glucans to recapitulate the architecture of β-glucan in the cell wall. The activation of human neutrophils by particulate yeast β-glucan is dependent on CR3 [18]. We also found that ligation of the particulate form of *C. albicans* and *C. glabrata* β-glucans to CR3 activated DC responses (figures 5 and 6). Additionally, particulate *Candida* β-glucans interacted with dectin-1 and CR3 in a different manner, which resulting in the differential immune responses (figures 5–7). These differences can be simply explained by the distinct signal transduction of dectin-1-dependent and CR3-dependent pathways. The ligation of β-glucans to dectin-1 mainly mediates downstream signalling via the Syk/CARD9 axis, while the ligation of β-glucans to CR3 induces a cellular response via the Src/ERK and Syk/PI3K pathways [28,57–59].

Treg, which play a critical role in immune suppression and enhance *Candida* pathogenicity, are greatly increased in disseminated invasive candidiasis caused by *C. albicans* [6]. The induction of Treg by *C. albicans* requires Toll-like receptor-2 and TRIF signalling in antigen-presenting cells [11,12]. In this study, we found that *C. glabrata* was also capable of Treg induction, but via the engagement of its β-glucan to CR3 on DCs (figures 1, 2 and 7). Although *C. glabrata*β-glucan activated dectin-1 and CR3 (figures 5 and 6), a binding assay suggested that *C. glabrata*β-glucan preferentially bound to CR3 (figure 10). The signal transduction via dectin-1 on DCs appeared to have less influence on Treg induction, while the signal from CR3 was probably dominant, which consequently led to Treg induction (figure 7*b*). One mechanism that may be involved in this Treg induction is via the PD-1/PD-L1 pathway [34,35]. This possibility is supported by our finding that ligation of *C. glabrata* β-glucan to CR3, but not dectin-1, induced high PD-L1 expression, and this expression was inhibited by CR3 blockade (figure 7*e,f*). The *C. glabrata*β-glucan-mediated immunomodulatory function of CR3 may result from the activation of CD11b [28]. A recent study showed that the immunomodulatory effect of soluble β-glucan depended on the stimulation of CR3, and this stimulation may be owing to the ligation of soluble β-glucan to the lectin site located on CD11b of CR3 [19,60]. Additionally, CD11b signalling can negatively regulate inflammatory signalling in immune cells [59,61] and the activation of CD11b in DCs can suppress the DC response and inhibit T-cell activation [62,63]. The interference of CD11b ligation in this study indicated immunomodulatory activity of *C. glabrata* β-glucan (figure 7).

The high IL-10 production induced by *C. glabrata* β-glucan may be attributed to the RORγt^+^ Foxp3^+^ Tr17. Previous studies have shown that RORγt^+^ Foxp3^+^ Tr17 are significant producers of IL-10 [64]. The development of Tr17 is dependent on IL-6/stat3 signalling [65]. Stimulation of BMDCs with *C. glabrata* β-glucan increased the production of IL-6 (figure 4*g*). However, in our study, the blockade of CR3 enhanced IL-6 production (figure 6*d*) while reducing the number of Foxp3^+^ Treg (figure 7*b*). Another line of evidence suggests that RORγt repressed IL-10 production [66], and loss of IL-10 led to increased RORγt expression [67]. Therefore, further study of this RORγt^+^ Foxp3^+^ Tr17 subpopulation in response to *C. glabrata* infection is necessary for the development of therapeutic applications.

In this study, we used GM-CSF and IL-4-derived BMDCs to examine DCs *in vitro*. This culture system is cost-effective, and primary BMDCs have shown better results than DC cell lines. However, GM-CSF-derived BMDCs consist of a mixed population of DCs and macrophages, as reported by Helft *et al*. [68]. Including IL-4 in GM-CSF-derived DC cultures has been shown to reduce the number of macrophages [68], indicating that DCs are likely the primary cell population in GM-CSF and IL-4-derived BMDCs in our system. Nevertheless, further research exploring DC responses to Candida β-glucan in alternative *in vitro* DC culture systems, such as FLT3L-derived BMDCs, may provide additional benefits and insights into DC-mediated anti-fungal immunity [69].

In conclusion, our findings suggest the underlying mechanisms responsible for inducing Treg by systemic *C. glabrata* infection. In this study, a single strain of each *Candida* species was selected for investigating the immune response related to inter-species variation of cell wall β-glucan. Nonetheless, intra-species diversity of cell wall β-glucan should be considered and further investigated to confirm the role of *C. glabrata* β-glucan. A better understanding of immune modulation by *C. glabrata* may lead to important contributions to the development of new immunotherapeutic strategies for treating invasive *C. glabrata* infection.

## 4. Material and methods

An extended description of the methods can be found in the electronic supplementary material.

### 4.1. Animals

Six- to seven-week-old female C57BL/6 mice were purchased from Nomura Siam International Co. (Bangkok, Thailand).

### 4.2. Cultivation of *Candida* yeasts

*Candida glabrata* (ATCC2001) and *C. albicans* (SC5314) were selected because these reference strains are used for quality control and antifungal drug susceptibility testing. Additionally, the cell wall glucans of both *Candida* strains have been well characterized [37,39,54]. All *Candida* yeasts were cultured in yeast peptone dextrose (YPD) agar (HiMedia Laboratories, Mumbai, India) at 30°C for 48 h. The yeast cells (5 × 10^6^ cells) from the colonies of each strain were subcultured in 15 ml of YPD broth at 30°C with shaking at 180 rpm for 8 h. Subsequently, *Candida* cultures were diluted to an OD_600_ of 0.1 and grown in 1.2 l of YPD broth medium at 30°C with shaking at 150 rpm for 13 h [39]. Under these culture conditions, all *Candida* species grow as budding yeast-like cells [70–72]. The morphology of all *Candida* yeasts was confirmed using bright field microscopy (Olympus BX50, Tokyo, Japan).

### 4.3. Preparation of heat-killed yeasts

Heat-killed yeasts were prepared as previously described [43]. *Candida* yeast cells were harvested and washed twice with sterile Dulbecco’s phosphate-buffered saline (DPBS; GIBCO, Thermo Fisher Scientific, NY, USA). The yeasts were then diluted to 1 × 10^8^ cells ml^−1^ in sterile DPBS and were inactivated by heating at 100°C for 10 min. The heat-killed yeast cells were collected by centrifugation and resuspended in RPMI 1640 (GIBCO, Thermo Fisher Scientific).

### 4.4. *Candida* mannan extraction

Cell wall mannans from *Candida* were extracted as previously described [43,73]. Briefly, 100 g of *Candida* yeast cell pellets were resuspended in 250 ml of citrate buffer (0.02 M, pH 7.0), and the yeast suspension was autoclaved at 121°C for 90 min. After cooling down, the supernatant was separated and collected by centrifugation, and the residual sediment was re-extracted with the same procedures. The supernatants from the first and second autoclaves were combined. Subsequently, an equal volume of Fehling’s solution was added, and the mixture was stirred at 4°C for overnight. The sediment of mannan–copper complex was collected by centrifugation and then dissolved in 6–8 ml of 3 N HCl. The solution was then added drop-wise into 100 ml of methanol–acetic acid (ratio: 8 : 1, v/v) to remove the copper. The mannan precipitate was collected by centrifugation and washed with HCl and methanol-acetic acid repeatedly until the precipitate became colourless. The mannan precipitate was dissolved in sterile water and further dialysed in sterile water. The concentration of mannan was determined using the phenol–sulfuric acid method [74]. Mannan was kept in the lyophilized form. All yeast culture and mannan extraction procedures were performed using endotoxin-free water and containers.

### 4.5. *Candida* β-glucan extraction

The preparation of *Candida* β-glucan microparticles, including extraction, depyrogenation and sterilization, was performed by following the protocol provided by Prof. David L. Williams (East Tennessee State University, Johnson City, TN, USA) [37,54]. Briefly, *Candida* yeasts were resuspended in 0.1 N NaOH and boiled at 100°C for 15 min. After cooling down, the residues were collected by centrifugation. The residue was resuspended in 0.1 N NaOH and the same procedure was repeated two more times (a total of three extractions). The harvested residues were boiled at 100°C in 2NH_3_PO_4_ for 15 min (three extractions) and then boiled at 100°C in acidic ethanol (1%v/v of H_3_PO_4_ in absolute ethanol) for 15 min (three extractions). The resulting residues were neutralized to a pH of 7.0. The β-glucan microparticles were washed three times with endotoxin-free water. Isolated β-glucan microparticles were depyrogenated in 250 mM NaOH and subsequently neutralized to a pH of 7.0 in 250 mM H_3_PO_4_. The β-glucans were washed in endotoxin-free water (three times) and sterilized by autoclaving at 121°C under 15 psi of pressure for 30 min. The β-glucans were stored in a lyophilized form until later use.

All yeast culture and β-glucan extraction procedures were performed using endotoxin-free water and containers. The three batches of extracted glucans were pooled and used for all experiments. The concentrations of β-glucans in all experiments are used as dry weight per volume (w/v).

### 4.6. Structure analysis by NMR spectroscopy

Chemical structure characterization was conducted using a JEOL JNM-ECZ500R/S1 spectrometer (JEOL, Peabody, MA, USA) operating at 500 MHz for ^1^H NMR and 126 MHz for ^13^C NMR. To perform *C. glabrata* β-glucan analysis, 50 mg of β-glucan was dissolved in 1.57 ml of DMSO-*d*_6_ and 0.08 ml of TFA-d_1_. Approximately 0.5 ml of the solution was then withdrawn to perform the NMR experiment at room temperature. NMR chemical shifts were referenced to the residual DMSO-*d*_6_ proton at δ 2.54 ppm and carbon at δ 40.45 ppm.

### 4.7. Scanning electron microscopy

*Candida* β-glucans were fixed in 2.5% glutaraldehyde in 0.1 M phosphate buffer (pH 7.2) at 4°C for 18 h. After fixation, the β-glucans were washed with sterile deionized water and were air dried. Subsequently, the β-glucans were processed for ultra-thin gold coating (JFC-1200; JEOL). The specimens were observed using scanning electron microscopy (Quanta250; FEI, Hillsboro, OR, USA) with 10 000× magnification.

### 4.8. Systemic *C. glabrata* infection and *ex vivo* re-stimulation

Mice were intraperitoneally administered dexamethasone (0.1 mg g^−1^ of body weight; Dexton-Vet, T. P. Drug Laboratories (1969), Bangkok, Thailand) 3 consecutive days before infection and at 5 days post-infection [39,75]. On day 0, the mice were intravenously inoculated with 5 × 10^6^, 10 × 10^6^ and 30 × 10^6^ of *C. glabrata* yeast cells in 100 µl of DPBS. In the control group, the mice received 100 µl of DPBS. On day 7 post-infection, serum and spleens were harvested from all control and infected mice.

Spleens were mechanically disrupted and red blood cells were removed using red blood cell lysis buffer (BioLegend, San Diego, CA, USA). Splenocytes were then washed in complete RPMI medium (RPMI 1640 containing 10% heat-inactivated fetal bovine serum (GIBCO), 2 mM Glutamax (GIBCO), 100 U ml^−1^ penicillin and 100 mg ml^−1^ streptomycin (GIBCO)). Splenocytes (2 × 10^6^ cells per sample) were stained for CD3, CD4, CD25 and Foxp3.

In the *ex vivo* re-stimulation assay, 200 µl of 10 µg ml^−1^ of purified anti-mouse CD3 antibody (clone 145-2C11; BioLegend) was coated on a 24-well plate at 4°C overnight. The anti-CD3 solution was then removed, and the coated wells were rinsed with culture medium twice. The splenocytes were then seeded at a concentration of 4 × 10^6^ cells per well in 1 ml of complete RPMI medium containing 55 µM 2-mercaptoethanol (2ME; GIBCO). Forty-eight hours after the stimulation, culture supernatants were collected for cytokine quantification, and the cells were harvested and stained for CD3, CD4, CD25, Foxp3 and IL-10.

### 4.9. *In vivo* immunization and *ex vivo* re-stimulation

In the scruff of the neck, mice were subcutaneously administered *C. glabrata* mannan or β-glucans (50 µg mannan or β-glucans per 1 g of body weight) mixed with OVA (Grade V; Sigma Aldrich, Saint Louis, MO, USA) (30 µg per mouse) in 200 µl of DPBS on days 0 and 7. The positive control mice were subcutaneously administered 200 µl of CFA and incomplete Freund’s adjuvant (IFA) mixed with OVA on days 0 and 7, respectively. The negative control mice received OVA in DPBS.

On day 14, the draining LNs (cervical, axillary and brachial LNs) were excised and digested with 300 U ml^−1^ of collagenase type IV (Sigma Aldrich) and 10 U ml^−1^ of DNase I (Sigma Aldrich) at 37°C with shaking at 1500 rpm for 30 min. The cells were washed and resuspended in complete RPMI medium.

In the *ex vivo* re-stimulation assay, LN cells (2 × 10^6^ cells) were cultured in 48-well plates in 0.5 ml of complete RPMI medium containing 55 µM 2ME in the presence of 250 µg ml^−1^ OVA. Forty-eight hours after the stimulation, the culture supernatant was collected for cytokine quantification, and the cells were harvested and stained for CD3, CD4, CD25 and Foxp3.

### 4.10. Generation and stimulation of BMDCs

BMDCs were generated *in vitro* as previously described [39,73]. Briefly, bone marrow cells were obtained from mouse femurs and tibias. The cells (1 × 10^6 ^1 ml^−1^) were seeded in 24-well plates and cultured in a complete RPMI medium supplemented with 10 ng ml^−1^ of recombinant murine GM-CSF (Peprotech, Rocky Hill, NJ, USA) and 10 ng ml^−1^ of recombinant murine IL-4 (Peprotech). The cells were incubated at 37°C under a humidified atmosphere containing 5% CO_2_ for 7 days. Half a volume of the media was replaced with the fresh media containing the cytokines every 2 days.

BMDCs were stimulated with heat-killed *Candida*, *Candida mannans* or β-glucans at various concentrations, as indicated in the figure legends. Twenty-four and 48 h after the stimulation, the culture supernatants were collected for cytokine measurements, and the cells were harvested for assessing DC maturation markers. Unstimulated BMDCs were used as the negative control.

### 4.11. Blockade of dectin-1 and CR3

BMDCs were pre-treated with a dectin-1 antagonist (soluble whole glucan particles; InvivoGen, San Diego, CA, USA) [39] or a purified anti-mouse/human CD11b antibody (clone M1/70; BioLegend) [76] for 2 h. The BMDCs were then stimulated with 25 µg ml^−1^ of *Candida* β-glucans. In the positive control, BMDCs were untreated or pre-treated with rat IgG for 2 h, and then the cells were stimulated with 25 µg ml^−1^ of *Candida* β-glucans. In the negative control, BMDCs were pre-treated with a dectin-1 antagonist or a purified anti-mouse/human CD11b antibody without *Candida* β-glucan stimulation.

Twenty-four and 48 h after the stimulation, the culture supernatants were collected for cytokine quantification, and the cells were harvested for assessing DC maturation markers.

### 4.12. *In vitro* direct co-culture of DCs and T cells

BMDCs were untreated or pre-treated with 25 µg ml of a dectin-1 antagonist or 10 µg ml^−1^ of a purified anti-mouse/human CD11b antibody for 2 h. The cells were subsequently stimulated with 25 µg ml^−1^ of *Candida* β-glucans for 24 h. The BMDCs were collected and washed with completed RPMI medium twice. The BMDCs (1 × 10^5^ cells) were co-cultured with T cells (1 × 10^6^) isolated from spleens of intact mice using immunomagnetic beads (Pan T Cell Isolation Kit II, mouse; Miltenyi Biotec, San Diego, CA, USA) at a DC : T-cell ratio of 1 : 10 in the presence of 30 ng ml^−1^ of soluble anti-mouse CD3 antibody (clone 145-2C11, BioLegend) in 48-well plates [31,32,39]. At 48 h, the culture supernatants were collected for cytokine quantification, and the cells were stained for CD3, CD4, CD25 and Foxp3.

### 4.13. Dectin-1 and CR3 binding assay

RAW264.7 cells were incubated with various amounts of *C. albicans* or *C. glabrata* β-glucan for 20 min, and the cells were washed twice. Subsequently, the cells were stained with fluorescent-tagged anti-dectin-1 mAbs or fluorescent-tagged anti-CD11b mAbs. The expression of dectin-1 and CR3 was determined by flow cytometry. The percentage binding was calculated by the following formula: 100 − (MFI of sample treated with β-glucan × 100 per MFI of untreated sample).

### 4.14. Flow cytometric analysis

BMDCs were stained with fluorochrome-tagged mAbs specific to mouse CD11c (clone N418), CD40 (clone 3/23), CD80 (clone 16-10A1), CD86 (clone GL-1), I-A/I-E (MHC class II) (clone M5/114.152) and PD-L1 (clone MIH7). LN cells, splenocytes and T cells were fluorochrome-tagged mAbs specific to mouse CD3 (clone 145-2C11), CD4 (clone GK1.5), CD25 (clone PC61) and mouse/human FoxP3 (clone FJK-16s). RAW264.7 cells were stained with fluorochrome-tagged mAbs specific to mouse dectin-1 (clone RH-1) and mouse/human CD11b (clone M1/70). All cells were incubated with Fc block (purified anti-mouse CD16/32, clone 93) to reduce non-specific binding before staining the cells with specific antibodies. All antibodies were obtained from BioLegend, except for the anti-FoxP3 antibody, which was obtained from eBioscience (San Diego, CA, USA). The stained cells were evaluated by flow cytometry (CytoFLEX; Beckman Coulter, San Diego, CA, USA), and the data were analysed using Kaluza Flow Analysis Software (Beckman Coulter).

To determine BMDCs and T cells, live cells were gated and the cells were acquired at 20 000 cells per sample. With regard to LN cells and splenocytes, live cells were gated and the cells were acquired at 50 000 cells per sample. Accordingly, the same electronic gate was applied to the same set of samples for flow cytometric acquisition and analyses.

### 4.15. Cytokine quantification

The supernatants were quantified by sandwich ELISA by following the manufacturer’s instructions. ELISA kits of mouse IFN-γ, IL-1β, IL-6, IL-12, IL-10, IL-17 and TNF-α were obtained from Biolegend, and the ELISA kit of mouse IL-23 was obtained from eBioscience. The absorbance was measured at 450 nm using a microplate reader (Biotek EPOCH2C, Agilent Technologies, Santa Clara, CA, USA).

### 4.16. Statistical analysis

All data are presented as the mean ± s.d. The data were statistically analysed using one-way analysis of variance with the post hoc Tukey HSD test, except for group comparison with continuous outcome variables shown in figure 3, which were analysed using two-way analysis of variance. All statistical analysis was performed using SPSS 22 software (IBM Corp., Armonk, NY, USA). Values of *p* < 0.05 were considered statistically significant.

## Data Availability

Supplementary data are available online [77].
